# The Expression and Related Clinical Significance of SIRT3 in Non-Small-Cell Lung Cancer

**DOI:** 10.1155/2017/8241953

**Published:** 2017-08-30

**Authors:** Guo-Cai Yang, Bi-Cheng Fu, Dong-Yang Zhang, Lu Sun, Wei Chen, Long Bai, Tong Gao, Hong-Guang Lu, Zhao-Yu Wang, Qiong-Qiong Kong, Lin Qiu, Hai Tian

**Affiliations:** ^1^Department of Thoracic and Cardiovascular Surgery, Second Affiliated Hospital of Harbin Medical University, Harbin, China; ^2^Lung Cancer Research Center, Zhoushan Hospital, Zhoushan, China; ^3^Key Laboratory of Myocardial Ischemia, Harbin Medical University, Ministry of Education, Harbin, China

## Abstract

**Objective:**

To examine the relationship between the Sirtuin-3 (SIRT3) expression and the clinical indicators/prognosis of patients with non-small-cell lung cancer (NSCLC).

**Methods:**

The mRNA level of SIRT3 was detected by real-time PCR, while the protein level was detected by Western blot and immunohistochemical staining. SPSS 16.0 software was used for statistical analysis.

**Results:**

The expression of SIRT3 was significantly higher in NSCLC tissue than in adjacent tissue. The SIRT3 level was correlated significantly with lymph node metastasis and clinical stage of NSCLC patients. Moreover, univariate analysis showed that the expression of SIRT3, tumor size, lymph node metastasis, degree of differentiation, and clinical stage were correlated with the prognosis of NSCLC patients. Multivariate analysis demonstrated that lymph node metastasis, the tumor size, and SIRT3 expression were independent prognostic factors for NSCLC patients.

**Conclusions:**

SIRT3 is associated with the development and progression of NSCLC. The SIRT3 expression can be used as an independent prognostic factor for NSCLC patients and help identify prognosis of NSCLC. Therefore, SIRT3 has the potential to become a new factor for prognosis prediction and personalized treatment of NSCLC.

## 1. Introduction

Lung cancer has become the most common cancer worldwide, which is the leading killer endangering public health in China [1, 2]. Non-small-cell lung cancer (NSCLC) including squamous carcinoma, adenocarcinoma, and large-cell carcinoma accounts for about 80% of lung cancer cases. However, the prognosis prediction and personalized treatment of the NSCLC are not satisfactory in clinic [3]. Therefore, the search for biomarkers of prognosis of NSCLC at the molecular level has great significance for developing individual treatment strategies.

SIRT3, a member of the silent information regulator (Sirtuins) family, participates in cell biological functions such as energy metabolism and cell aging by regulating mitochondrial function, and it is closely related to tumor development and progression [4–6]. However, there is still a controversy whether SIRT3 plays as an oncogene or a suppressor gene in tumors, which becomes conflicting results reported in different types of tumors [7–9] Besides, the expression and role of SIRT3 in lung cancer have not been generally acknowledged. Therefore, in this study, we analyzed the relationship between SIRT3 expression and clinical characteristics of the NSCLC patients by detecting the mRNA and protein levels of SIRT3 in both tumor tissue and adjacent normal lung tissue and then defined the relationship between the expression of SIRT3 and the outcome of patients with NSCLC.

## 2. Materials and Methods

### 2.1. General Information

40 fresh surgically resected NSCLC tissue samples and matched adjacent tissue (more than 3 cm from tumor tissue) were collected during January 2015 to March 2015 and rapidly frozen at −80°C for subsequent determination of SIRT3 mRNA and protein levels.

In addition, 131 matched pairs of archived paraffin samples from primary NSCLC patients were collected during January 2009 to December 2010 for immunohistochemical analysis. All patients had been pathologically diagnosed as primary NSCLC and did not receive any anticancer therapy before surgery, there were 73 men and 58 women. Patients were 35 to 82 years old at the time of surgical resection (median age 58 years), including 80 cases with age ≥ 60 years and 51 cases with age < 60 years. There were 92 patients with lymph node metastasis and 39 without lymph node metastasis. According to the WHO histological classification criteria (2015), there were 39 cases of squamous cell carcinoma and 92 cases of adenocarcinoma; 33 well-differentiated cases, 62 moderately differentiated cases, and 36 poorly differentiated cases. According to the TNM staging criteria revised by the International Union Against Cancer (UICC) (2009), there were 11 cases of stage 0, 45 cases of stage I, 53 cases of stage II, and 22 cases of stage III.

The use of all samples was approved by the ethics committee of Zhoushan Hospital. The informed consent was obtained from all patients. Patients were followed up through telephone call and outpatient visit. Survival time was defined as the time period from the date of surgery to the death due to recurrence/metastasis. The last follow-up was made in December 2015. One patient failed to be followed up.

### 2.2. Methods

#### 2.2.1. Gene Expression of SIRT3 in Fresh Paired NSCLC Tissue and Adjacent Tissue

Samples were ground, and total RNAs were extracted using TRIzol according to the manufacturer's instructions. RNAs were treated with DNase I and then reverse transcribed to cDNAs using AccuPower RocketScript RT PreMix (Bioneer, USA). Real-time PCR was performed to determine the relative expression of SIRT3 gene with AccuPower 2×GreenstarqPCR Master Mix (Bioneer) on a Thermal cycler (Bio-Rad, USA). The mRNA levels of SIRT3 in NSCLC tissue and adjacent tissue were calculated using the 2^−ΔΔCT^ method. *β*-Actin served as the internal control. PCR primers were designed and synthesized by Bioneer. Primer sequences are listed in Table 1.

#### 2.2.2. Protein Expression of SIRT3 in Fresh Paired NSCLC Tissue and Adjacent Tissue

The expression of SIRT3 protein was determined by Western blot. Total proteins were extracted using RIPA (Solarbio, China) and protease inhibitor (Roche, USA). Protein concentrations were determined using the BCA kit (Beyotime Biotechnology, China). The antibodies used for Western blot included SIRT3 antibody (Cell Signaling Technology, catalogue number 2627, diluted 1 : 1000), *β*-actin monoclonal antibody (Zhongshan Golden Bridge, diluted 1 : 500), horseradish peroxidase-labeled goat anti-rabbit IgG (Zhongshan Golden Bridge, diluted 1 : 8000), and horseradish peroxidase-labeled goat anti-mouse IgG (Zhongshan Golden Bridge, diluted 1 : 8000). The gray values of protein bands were evaluated using ImageJ. The ratio of SIRT3/*β*-actin was used as the relative level of protein expression.

#### 2.2.3. Protein Expression of SIRT3 in Paired Paraffin-Embedded NSCLC Tissue and Adjacent Tissue

Immunohistochemical staining was performed using the Max Vision two-step technique. Paraffin samples were serially sectioned to a thickness of 4 *μ*m, dewaxed, immersed into water, antigen-retrieved for 2-3 min, and blocked with hydrogen peroxide for 10 min. Tissue slices were incubated with primary antibody at 37°C for 60 min and then with secondary antibody for 30 min. The slices were then visualized using DAB, counterstained with hematoxylin, dehydrated, cleared, and mounted.

For evaluating the immunohistochemical results, the expression of SIRT3 was scored by two senior pathologists according to the double-blind method. The expression of SIRT3 was localized in the cytoplasm and nucleus, which appeared as yellow or brown particles by immunohistochemical staining. The number of positive cells was counted using the secondary scoring method. Samples treated with PBS buffer instead of primary antibody were used as the negative control. Five fields (200x) were randomly selected from each slice, and 100 cells (tumor cells or alveolar epithelial cells) were counted in each field. For the semiquantitative evaluation of SIRT3 expression, we used the Allred scoring method [10, 11]: First, the positive level of SIRT3 expression was devided into five stages (0–4 points), corresponding to the positive rate of ≤5%, 6–25%, 26–50%, 51–75%, and ≥76%. Second, the intensity of immunohistochemical staining was divided into four levels (0–3 points), corresponding to no coloring, pale yellow granules, yellowish brown granules, and dark brown granules. The final scores were added using the above two steps. We defined 0-1 point as negative expression (−), 2-3 points as weak expression (+), 4-5 points as moderate expression (++), and 6-7 points as strong expression (+++).

### 2.3. Statistical Analysis

SPSS 16.0 software was used for statistical analysis. The difference of SIRT3 expression levels in NSCLC tissue and adjacent tissue was compared using paired rank tests. The correlation between SIRT3 expression and various clinical indicators was analyzed using nonparametric independent samples rank sum test. The correlation between clinical and pathological factors and outcome of NSCLC patients after surgery was assessed using univariate and multivariate analyses. The log-rank method was adopted for univariate analysis, and the COX hazards regression model was employed for multivariate analysis. *p* < 0.05 was considered statistically significant.

## 3. Results

### 3.1. The Gene and Protein Expression of SIRT3 in Fresh Paired NSCLC Tissue and Adjacent Tissue

**Figure 1:**
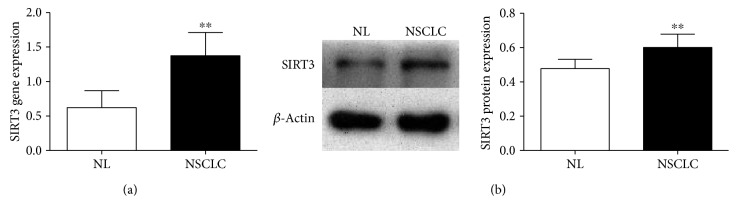
The gene and protein expression of SIRT3 in NSCLC and adjacent tissue. (a) The mRNA expression of SIRT3 was higher in NSCLC tissue than in adjacent tissue. (b) The protein expression of SIRT3 was higher in NSCLC tissue than in adjacent tissue *n* = 6, ^∗∗^*p* < 0.01.

To explore the relationship between SIRT3 and NSCLC, the mRNA and protein expression of SIRT3 were detected in NSCLC and adjacent tissue using real-time PCR and Western Blot. The results showed that the expression levels of SIRT3 were significantly higher in NSCLC tissue than in adjacent tissue (Figures 1(a) and 1(b)), suggesting SIRT3 may be associated with NSCLC.

### 3.2. The Protein Expression of SIRT3 in Paired Paraffin-Embedded NSCLC Tissue Samples and Adjacent Tissue

**Figure 2:**
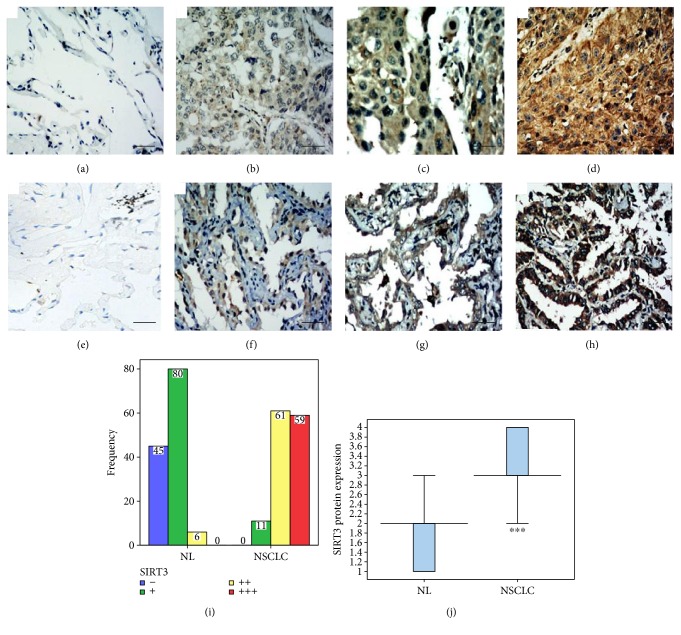
The expression of SIRT3 in NSCLC and adjacent tissue (bar = 50 *μ*m, 200x). (a) Negative expression (−) in normal lung tissue adjacent to squamous cell carcinoma. (b) Weak expression (+) in lung squamous cell carcinoma. (c) Moderate expression (++) in lung squamous cell carcinoma. (d) Strong expression (+++) in lung squamous cell carcinoma. (e) Negative expression (−) in normal lung tissue adjacent to lung adenocarcinoma. (f) Weak expression (+) in lung adenocarcinoma. (g) Moderate expression (++) in lung adenocarcinoma. (h) Strong expression (+++) in lung adenocarcinoma. (i, j) The expression of SIRT3 was higher in NSCLC tissue than in adjacent tissue (*Z* = −10.146, ^∗∗∗^*p* < 0.001).

To further verify the correlation, we determined the protein expression of SIRT3 in paired paraffin-embedded NSCLC and adjacent tissue. The protein expression of SIRT3 could be detected in NSCLC cells by immunohistochemical staining. However, SIRT3 was at varying levels in the cytoplasm and nuclei, mainly concentrated in the cytoplasm. SIRT3 appeared as diffuse or scattered brown particles (Figures 2(a), 2(b), 2(c), 2(d), 2(e), 2(f), 2(g), and 2(h)). The results of 131 matched samples of NSCLC and adjacent tissue were showed in Figure 2. The SIRT3 expression of NSCLC tissues showed that strong positive was in 59 cases (45%), moderate in 61 cases (46.6%), weak in 11 cases (8.4%), and negative in 0 case (0%) (Figures 2(i) and 2(j)). The SIRT3 expression of adjacent tissues showed that strong positive was in 0 case (0%), moderate in 6 cases (4.6%), weak in 80 cases (61.1%), and negative in 45 cases (34.3%) (Figures 2(i) and 2(j)). The expression of SIRT3 was higher in NSCLC tissue than in adjacent tissue, except one case where the expression level of SIRT3 was the same in NSCLC and adjacent tissue.

### 3.3. The Correlation between SIRT3 Expression and Clinical Indicators

The secondary scoring method was used to evaluate the intensity of SIRT3 expression between tumor and adjacent tissue (0–2, low expression; 3–5, moderate expression; and 6-7, high expression). We used nonparametric independent samples rank sum test to analyze the correlation between the SIRT3 expression and clinical indicators. The results showed that the expression level of SIRT3 was correlated significantly with lymph node metastasis (Table 2) and clinical stage of NSCLC patients (Table 2), but no relationship with gender, age, histological type, tumor size, tumor site, pleural invasion, and degree of differentiation.

### 3.4. The Correlation between Clinical Indicators/SIRT3 Expression and Prognosis

**Figure 3:**
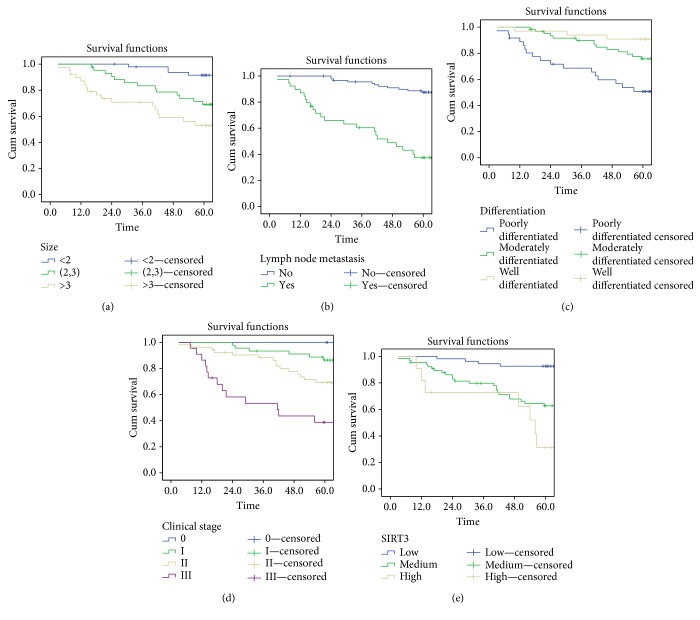
The correlation between clinical indicators/SIRT3 expression and survival time. (a) The correlation between tumor size and survival time. (b) The correlation between lymph node metastasis and survival time. (c) The correlation between the degree of differentiation and survival time. (d) The correlation between clinical stage and survival time. (e) The correlation between SIRT3 expression level and survival time.

To further explore the relationship between clinical indicators/SIRT3 expression and prognosis, the univariate analysis was used. The results showed that gender, age, histological type, tumor site, and pleural invasion were not correlated with the prognosis of NSCLC patients. Meanwhile, the tumor sizes, lymph node metastasis, degree of differentiation, and clinical stage were significantly correlated with survival time in patients with NSCLC (Table 3, Figures 3(a) and 3(d)). NSCLC patients with a smaller or high-differentiated tumor had a longer survival time, while patients with lymph node metastasis or more advanced tumor stage had a shorter survival time.

The expression level of SIRT3 was also significantly correlated with survival time of NSCLC patients (Table 3, Figure 3(e)), demonstrating that NSCLC patients with higher expression of SIRT3 had a shorter survival time.

For above five indicators that were found to be statistically significant in the univariate analysis, we further used the Cox proportional hazards regression model to explore whether these indicators could be independent predictors of survival time. The results showed that the presence or absence of lymph node metastasis and the tumor size were independent predictors of survival time. More importantly, the expression level of SIRT3 could serve as an independent prognostic factor for NSCLC patients (Table 4).

## 4. Discussion

Tumor development and progression is a complex process that involves various factors and multiple stages. Cellular and genetic changes occur during malignant transformation of cells, ultimately leading to the formation of tumor cells [12].

Sirtuins have various enzymatic activities and are involved in the regulation of a variety of cellular functions, including metabolism, aging, cancer, and stress responses [13]. SIRT3, a member of the Sirtuin family, resides in the mitochondria and acts as a mitochondrial deacetylase. SIRT3 which controls the activation and deactivation of many important enzymes is related to the levels of reactive oxygen species (ROS) in the mitochondria and offers significant protection against aging and cancer [13–16]. There are evidences that SIRT3 is involved in tumor development and progression by regulating cell apoptosis, genomic instability and mutation, cell proliferation and signal transduction, cell energy metabolism, development of inflammation, and tumor invasion [17, 18]. Until now, it has been found that SIRT3 is closely implicated in a variety of tumors, such as human oral cancer [7], esophageal cancer [19], colon cancer [20], liver cancer [21], breast cancer [9], melanoma [22], and thyroid cancer [23]. However, the relationship between SIRT3 and NSCLC remains unclear.

In this study, we found that the expression of SIRT3 was significantly higher in NSCLC tissue than in adjacent tissue for the first time. More importantly, we initially demonstrated that SIRT3 expression levels are closely associated with survival time of NSCLC patients, that is, the higher expression of SIRT3 leading the shorter survival time in NSCLC patients. Similar results are also reported in other types of cancer, such as oral cancer [7] and esophageal cancer [19]. However, it remains controversial whether SIRT3 acts as a tumor promoter or a tumor suppressor in tumorigenesis, because there were conflicting results in colon cancer [20], liver cancer [21], and breast cancer [9]. We thought that such a discrepancy may be caused by different regulatory role of SIRT3 in different types of cancer. Therefore, it deserves our further study on the effect and mechanism of SIRT3 in cancer.

As a clinical study, we paid more attention to the clinical significance of SIRT3. Nowadays, for patients with NSCLC, the presence or absence of lymph node metastasis and the tumor size are the most reliable and widely accepted predictors of survival. Nevertheless, many patients with the same clinical stage have totally different outcomes. This phenomenon indicates that the current predictor might be insufficient to fully evaluate and predict clinical outcome. In this study, we found that the expression level of SIRT3, the presence or absence of lymph node metastasis, and the tumor size were independent predictors of survival time. Furthermore, SIRT3 expression was correlated with lymph node metastasis and clinical stage. The results above provide useful information for predicting the biological behaviors of NSCLC by analyzing the expression of SIRT3. Therefore, we speculate that SIRT3 is involved in the development and progression of NSCLC.

However, our study is not without its limitations. In our study, much remains to be learnt about the specific role and potential mechanisms responsible for the effects of SIRT3 in the NSCLC and a more comprehensive exploration is necessary in the future. What is more, our further studies should, firstly, focus on the use of SIRT3 to predict NSCLC patient survival and to select the patients who will benefit from specific treatments and, secondly, focus on the development of targeted SIRT3 therapies to design personalized treatments. We hope that a comprehensive network of SIRT3 treatment and NSCLC prognosis evaluation system could be established in the near future.

In summary, SIRT3 can serve as an independent predictor of prognosis in patients with NSCLC. The expression level of SIRT3 can help identify NSCLC subgroups with a good or poor prognosis. On the other hand, the further research is needed to reveal the mechanism of SIRT3 in NSCLC.

## Figures and Tables

**Table 1:** Primer sequences for real-time PCR.

Primer	Sequence
SIRT3-F	5′-GCATCCCTGCCTCAA AGC-3′
SIRT3-R	5′-CGTCAGCCCGAATGTCCTC-3′
*β*-Actin-F	5′-CCCAGCACAATGAAGAT CAAGATCAT-3′
*β*-Actin-R	5′-ATCTGCTGGAAGGTGTACAGCGA-3′

**Table 2:** The correlation between SIRT3 expression levels and clinical indicators.

Clinical indicator	*N*	SIRT3 expression levels			*χ* ^2^ or *Z*	*p*
		Low	Medium	High		
Sex						
Male	73	30	36	7	0.317	0.853
Female	58	24	30	4		
Age						
≥60	80	33	41	6	0.153	0.878
<60	51	21	25	5		
Pathological type						
SCC	39	18	16	5	2.587	0.274
ACA	92	36	50	6		
Tumor size						
≤2	49	21	26	2	3.478	0.176
2-3	41	19	20	2		
≥3	41	14	20	7		
Tumor site						
Left lung	52	20	28	4	0.416	0.812
Right lung	79	34	38	7		
Lymphatic metastasis						
Yes	39	9	23	7	11.283	0.004
No	92	45	43	4		
Pleural invasion						
Yes	41	16	22	3	0.280	0.869
No	90	38	44	8		
Degree of differentiation						
Low	36	14	16	6	1.865	0.394
Medium	62	24	34	4		
High	33	16	16	1		
Clinical stage						
0	11	6	5	0	8.766	0.033
I	45	23	20	2		
II	53	20	29	4		
III	22	5	12	5		

**Table 3:** Univariate analysis of the correlation between clinical indicators/SIRT3 expression and prognosis.

Clinical indicator	*N*	5-year survival rate (%)	Chi-square	Log-rank test (*p*)
SIRT3 expression level				
Low	54	87.0	20.525	0.000
Medium	66	56.1		
High	11	27.3		
Sex				
Male	73	64.4	0.800	0.371
Female	58	69.0		
Age				
≥60	80	65.0	0.515	0.473
<60	51	68.6		
Pathological type				
SCC	39	56.4	2.791	0.095
ACA	92	70.7		
Tumor size				
≤2	49	83.7	10.972	0.004
2-3	41	60.5		
≥3	41	51.3		
Tumor site				
Left lung	52	67.1	0.364	0.546
Right lung	79	65.4		
Lymphatic metastasis				
Yes	39	41.0	28.188	0.000
No	92	77.2		
Pleural invasion				
Yes	41	70.7	0.202	0.653
No	90	64.4		
Degree of differentiation				
Low	36	50.0	10.952	0.004
Medium	62	72.6		
High	33	72.7		
Clinical stage				
0	11	100	16.092	0.001
I	45	75.6		
II	53	62.3		
III	22	40.9		

**Table 4:** Cox multivariate analysis of the correlation between significant indicators and survival time.

Clinical indicator	Relative risk	95% confidence interval	*p* value
Tumor size	1.531	1.026–2.283	0.037
Lymphatic metastasis	3.018	1.613–5.630	0.001
Degree of differentiation	0.937	0.185–0.791	0.808
Clinical stage	0.885	0.494–1.586	0.681
SIRT3 expression levels	2.200	1.383–3.500	0.001

## References

[1] Chen W., Zheng R., Baade P. D. (2016). Cancer statistics in China, 2015. *CA: a Cancer Journal for Clinicians*.

[2] Miller Y. E. (2005). Pathogenesis of lung cancer: 100 year report. *American Journal of Respiratory Cell and Molecular Biology*.

[3] Reungwetwattana T., Weroha S. J., Molina J. R. (2012). Oncogenic pathways, molecularly targeted therapies, and highlighted clinical trials in non-small-cell lung cancer (NSCLC). *Clinical Lung Cancer*.

[4] Bhat T. A., Kumar S., Chaudhary A. K., Yadav N., Chandra D. (2015). Restoration of mitochondria function as a target for cancer therapy. *Drug Discovery Today*.

[5] Haigis M. C., Guarente L. P. (2006). Mammalian sirtuins—emerging roles in physiology, aging, and calorie restriction. *Genes & Development*.

[6] McDonnell E., Peterson B. S., Bomze H. M., Hirschey M. D. (2015). SIRT3 regulates progression and development of diseases of aging. *Trends in Endocrinology and Metabolism*.

[7] Alhazzazi T. Y., Kamarajan P., Joo N. (2011). Sirtuin-3 (SIRT3), a novel potential therapeutic target for oral cancer. *Cancer*.

[8] Alhazzazi T. Y., Kamarajan P., Verdin E., Kapila Y. L. (2011). SIRT3 and cancer: tumor promoter or suppressor?. *Biochimica et Biophysica Acta*.

[9] Desouki M. M., Doubinskaia I., Gius D., Abdulkadir S. A. (2014). Decreased mitochondrial SIRT3 expression is a potential molecular biomarker associated with poor outcome in breast cancer. *Human Pathology*.

[10] Takahashi Y., Sawada T., Akahane T. (2017). Monocyte chemoattractant protein 1 expression and proliferation in primary central nervous system lymphoma. *Oncology Letters*.

[11] Wei Q., Huang X., Fu B. (2015). IMP3 expression in biopsy specimens of colorectal cancer predicts lymph node metastasis and TNM stage. *International Journal of Clinical & Experimental Pathology*.

[12] Croce C. M. (2008). Oncogenes and cancer. *The New England Journal of Medicine*.

[13] Weir H. J., Lane J. D., Balthasar N. (2013). SIRT3: a central regulator of mitochondrial adaptation in health and disease. *Genes & Cancer*.

[14] Finley L. W., Haigis M. C. (2012). Metabolic regulation by SIRT3: implications for tumorigenesis. *Trends in Molecular Medicine*.

[15] Shulga N., Pastorino J. G. (2010). Ethanol sensitizes mitochondria to the permeability transition by inhibiting deacetylation of cyclophilin-D mediated by sirtuin-3. *Journal of Cell Science*.

[16] Schumacker P. T. (2011). SIRT3 controls cancer metabolic reprogramming by regulating ROS and HIF. *Cancer Cell*.

[17] Guarente L. (2013). Introduction: sirtuins in aging and diseases. *Methods in Molecular Biology*.

[18] Hanahan D., Weinberg R. A. (2011). Hallmarks of cancer: the next generation. *Cell*.

[19] Zhao Y., Yang H., Wang X., Zhang R., Wang C., Guo Z. (2013). Sirtuin-3 (SIRT3) expression is associated with overall survival in esophageal cancer. *Annals of Diagnostic Pathology*.

[20] Liu C., Huang Z., Jiang H., Shi F. (2014). The sirtuin 3 expression profile is associated with pathological and clinical outcomes in colon cancer patients. *BioMed Research International*.

[21] Zhang Y. Y., Zhou L. M. (2012). Sirt3 inhibits hepatocellular carcinoma cell growth through reducing Mdm2-mediated p53 degradation. *Biochemical and Biophysical Research Communications*.

[22] George J., Nihal M., Singh C. K., Zhong W., Liu X., Ahmad N. (2016). Pro-proliferative function of mitochondrial Sirtuin deacetylase SIRT3 in human melanoma. *The Journal of Investigative Dermatology*.

[23] Shackelford R., Hirsh S., Henry K., Abdel-Mageed A., Kandil E., Coppola D. (2013). Nicotinamide phosphoribosyltransferase and SIRT3 expression are increased in well-differentiated thyroid carcinomas. *Anticancer Research*.

